# Modeling the Dynamics of Electric Field-Assisted Local Functionalization in Two-Dimensional Materials

**DOI:** 10.3390/ma19010204

**Published:** 2026-01-05

**Authors:** Fernando Borrás, Julio Ramiro-Bargueño, Óscar Casanova-Carvajal, Alicia de Andrés, Sergio J. Quesada, Ángel Luis Álvarez

**Affiliations:** 1 Escuela de Ingeniería de Fuenlabrada, Universidad Rey Juan Carlos, 28942 Fuenlabrada (Madrid), Spain; f.borras.2019@alumnos.urjc.es (F.B.); julio.ramiro@urjc.es (J.R.-B.); 2 Centro de Tecnología Biomédica, Campus de Montegancedo, Universidad Politécnica de Madrid, 28223 Madrid, Spain; oscar.casanova@ctb.upm.es; 3 Departamento de Ingeniería Eléctrica, Electrónica, Automática y Física Aplicada, Escuela Técnica Superior de Ingeniería y Diseño Industrial ETSIDI, Universidad Politécnica de Madrid, 28040 Madrid, Spain; 4 Instituto de Ciencia de Materiales de Madrid, Consejo Superior de Investigaciones Científicas, Cantoblanco, 28049 Madrid, Spain; ada@icmm.csic.es; 5 Independent Researcher, 23006 Jaén, Spain; quesada@quesada-consulting.es

**Keywords:** 2D semiconductors, thin films, local anodic oxidation, numerical modeling, finite element calculation

## Abstract

Electric field-assisted local functionalization of materials is a resist-free technique generally applied at the nanoscale, which has been understood within the paradigm of the water meniscus. Using a home-made prototype the authors applied this technique at scales compatible with the biosensor industry (tens of microns). However, interpreting these results requires a different paradigm. The expansion of the oxidized region over time in two-dimensional materials under a localized electric field is modeled from first physical principles. Boltzmann statistics is applied to the oxyanion incorporation at the perimeter of the oxidized zone, and a new general relation between oxide radius and time is formulated. It includes the reduction in the energy barrier due to the field effect and its dependence on the oxide radius. To gain insight into this dependence whatever the layers structure, 2D material involved, or electrical operating conditions, simple structures based on multilayer stacks representing the main constituents are proposed, where the Poisson equation is solved using finite element calculations. This enables to derive energy barriers for oxyanion incorporation at varying spot radii which are consistent with those resulting from fitting experimental data. The reasonable agreement obtained provides researchers with a new tool to predict the evolution of local functionalization of 2D layers as a function of the following fabrication parameters: time, applied voltage, and relative humidity, solely based on materials properties.

## 1. Introduction

The process of binding small reactive molecules to a suitable substrate is a crucial step in the manufacture of biosensors. This process subsequently enables the attachment of bioreceptors capable of detecting specific biomarkers through chemical bonding. The spatial control of this process in different regions of the same layer may eventually enable the simultaneous detection of several analytes in a single sample, which is one of the holy grails in biosensing technology. To enhance sensitivity, commercial devices use to need functionalization over areas from hundreds to thousands of µm^2^ [1,2]. Scaling up to such large areas is a challenge regarding performance and speed of functionalization.

Local anodic oxidation (LAO) is a resist-free technique that is among the most widely used for the functionalization of two-dimensional (2D) materials, such as graphene. It affords covalent bonding, which confers robustness against subsequent chemical reactions, and allows positional control [3]. The oxidation process in bulk materials was originally formulated by Cabrera and Mott [4]. Their model addresses the problem that the first oxide layer covers the target material and, consequently, during subsequent oxidation oxygen must migrate from the surface to the depths of the oxide, namely towards the oxide-material interface. How oxygen atoms make that trip and the force driving the process are aspects considered by Cabrera and Mott’s model [4]. In the absence of a local external electric field, the driving force is the internal electric field resulting from the difference in electrochemical potential between the material-oxide interface and that at oxide-air. In this case, oxygen must somehow travel through the oxide layer, which is formulated by a thermally activated hoping transport, according to the principles proposed by Verwey, Dewald, and others [5,6]. That model was subsequently adapted to the LAO performed by a scanning probe technique, in which the process is locally enhanced by an electric field in the small region below a probe. Additionally, the previous formulations were improved to incorporate space charge effects [7,8,9] and layer strain [10].

During LAO the localized electric field speeds up the oxidation in different ways [11,12,13,14,15], of which the most relevant is the promotion of water condensation just within the small gap between the probe tip and sample [11,12]. In environments of low values of relative humidity (RH), e.g., RH < 50%, this water bridge confines the electrochemical reaction exclusively to the region beneath the probe tip. Consequently, oxidation operates in a regime limited by the supply of oxyanions. This has been exploited to obtain nanoscale patterns by utilizing very sharp tips. Thus, the presence of the water meniscus has become the predominant paradigm to explain LAO at the nanoscale over the past three decades [10,11,12]. A number of studies have focused on the water meniscus shape [16,17,18] as well as on mathematical formulations of the oxide growth in the vertical direction (height) over time within the volume provided by this water bridge. [19,20,21] For a comprehensive overview of the existing models, please refer to [8]. However, for moderate to high RH values (e.g., RH > 70%), when a significant wetting layer is created over the material, it has been observed that the oxidation progresses in the surface plane up to distances orders of magnitude greater than those in the vertical direction towards the interior of the sample [22,23]. Experimental studies on Si and MoSe_2_ flakes have confirmed that the oxidized region in bulk materials exhibits a distinctive “UFO” shape over time, slowly growing in height just below the probe, and much faster in the lateral dimension [10,14,15,24]. Therefore, the horizontal or lateral progress of oxidation appears to follow a different and independent mechanism. Within the water meniscus paradigm, the lateral expansion of oxidation is a complex phenomenon influenced by the electric field distribution within the water bridge and wetting layer, together with the initial charge density formation and the subsequent lateral diffusion of ionic species for a set of different reactions [21,25]. Mathematical formulations have so far employed empirical fits to experimental data [25,26,27], rather than universally applicable first-principles equations. Table 1 provides a summary of various empirical models that have been utilized.

The progress of LAO in two-dimensional (2D) layers exhibits characteristics that differ from those in the bulk (3D). Specifically, it does not imply the diffusion or migration of oxygen through the layer, which is a fundamental aspect in bulk oxidation. In this case, oxidation can be interpreted exclusively as the progressive incorporation of oxyanions at the perimeter of the oxidized zone. A detailed examination of the electrostatic characteristics along this perimeter suggests an approach that has not been previously explored to explain the temporal evolution of LAO in 2D. With this in mind, more attention must be paid to the electric field at the edge of the oxidized region, rather than just below the probe, as was traditionally done by the water meniscus paradigm. In fact, the water meniscus paradigm is relatively effective in explaining the widening of the initial oxidized zone with increasing applied voltage. However, when a homogeneous layer is formed above the 2D material because of a high RH, it fails to explain the progressive expansion of this zone with the exposure time, which is an important manufacturing parameter. As a result, the models currently in use to describe the evolution of the oxidized region diameter over time are empirical, rather than based on first physical principles (Table 1).

Based on these concepts, the authors proposed the general trends of a physical model that allows predicting fundamental aspects of the oxidation dynamics in 2D materials [28]. In that work, a preliminary formulation of both the expansion of oxide over time and the corresponding current during the process were suggested [28]. This was made possible by a unique instrumental prototype, which enabled the large-scale production of oxidation with positional control, where individual spots of thousands µm^2^ were performed at once, in intervals < 100 ms [29].

In this work, we provide a comprehensive description of the complete model, incorporating all pertinent fabrication parameters as follows: applied voltage, time, and relative humidity. This predictive nature is of great interest for establishing the operating conditions necessary to functionalize 2D materials, enabling high-speed control of extensive functional regions within the channel of field-effect transistor-based biosensors. We have replaced previously employed approaches in [28] with more accurate functions and have verified the model’s validity for both graphene and transition metal dichalcogenide (TMD) thin films.

## 2. Materials and Methods

The ability to control the position of the probe, and consequently the oxidized zones, by using automated steppers is one of the key features of LAO technology, which makes it particularly suitable for distributed biosensors. The instrument to perform LAO over extensive areas (rate of up to 10^4^ µm^2^/s) is equipped with 3 electromechanical linear stages developed by PI (Physik Instrumente, Karlsruhe, Germany) [29]. The system operates in contact mode without force feedback mechanism, so a commercial spring-probe (Tecon, Negrar di Valpolicella (VR), Italy) with a spring constant of about 300 N·m^−1^ is used to damp contact and avoid damage on the sample. The diameter at the apex was about 12 µm. The probe spring is compressed a maximum of 2 µm during the experiments. We note that for this compression, the exerted pressure is theoretically so small (10^5^–10^7^ Pa) that no mechanical damage could be detected on graphene layers, which is consistent with the high intrinsic strength reported for graphene (130 GPa) [30]. The implemented motion control allows automatized tilt correction upon previous mapping of the surface plane, so the force exerted on the sample may be kept constant during operation over large areas (up to mm^2^).

The oxidized spots on graphene shown in the following section were conducted with a voltage of −30V at the probe and RH conditions of 92% to ensure a uniform wetting layer. The precision thermo-hygrometer sensor is a SHT45-AD1B model by Sensirion (Stäfa, Switzerland) (±1.0% RH, in a range 0–100% RH) which is located few mm far from the sample for a reliable RH measurement. The exposure time for both oxidation and reduction was 240 ms. The most stable and reproducible results were obtained from CVD graphene single layers transferred onto quartz, although graphene on SiO_2_/Si:p+ was also studied. Both types of samples were supplied by the company Graphenea (San Sebastian, Spain).

Optical images were captured by differential interference contrast (DIC) microscopy, using a Leica DMR microscope provided with a DFC320 digital camera by Leica (Wetzlar, Germany). Micro-Raman spectroscopy was carried out at room temperature using the 488 nm line of an Ar+ laser. The light was focused and recorded through an Olympus microscope of high numerical aperture (NA = 0.95) allowing a spatial resolution of 0.7 µm.

To construct the electrostatic model that represents the experimental layer stack, and to solve the conventional Poisson equation with boundary conditions by finite element calculations throughout the proposed structure, the “Semiconductors” module of the software COMSOL Multiphysics (Version 4.4) has been utilized [31]. The least-square fittings of experimental data to the proposed models used a quasi-Newton method with a finite-difference gradient, programmed in FORTRAN 90 language within the Microsoft Developer Studio software (© Microsoft Corporation 94-95, Redmont, WA, USA).

## 3. Results

### 3.1. Operating Framework for Obtaining Electrical Magnitudes

The water meniscus paradigm for the operation at the nanoscale is based on the experimental detection of a water bridge formed between sample and the tip of an atomic force microscope [17,32]. It is justified by the significant enhancement of capillarity that occurs when an electric field is applied, and it has been mathematically modeled by calculations that consider the electric fields between tips with varying geometries and a metallic plane [33,34]. For a sharp conical probe with radial symmetry, the lateral decay of this field is very fast beyond the tip, scaling approximately as (r/r0)^−n^ (where r0 is the tip radius, and n = 3). Furthermore, the dielectric water bridge produces a lensing effect on the field lines, resulting in tighter confinement in the nearer region below the tip and a steeper lateral decay.

However, three observations indicate that this view is too simple to explain the local anodic oxidation of 2D materials when a consolidated wetting layer is present: (i) those calculated field distributions represent a static view of the process, whereas oxidation can spread gradually over time to long distances (up to more than ten times the tip radius in graphene, at moderate operating voltages < 40 V) [28]. (ii) Once oxidation begins, the material electrical properties undergo a dramatic change from conductive to insulating. This also affects the electric field distribution, which renders the above-mentioned models invalid and only suitable for describing the conditions during the pre-processing stage. (iii) Since the expansion progresses from the edge of the oxidized region, it is more appropriate to focus on the electric field at this edge and not on the small vertical gap between probe and sample.

**Figure 1:**
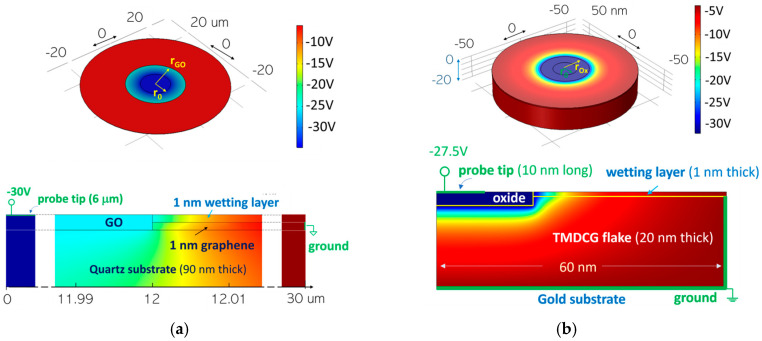
Layer structures to obtain electrical properties by finite element calculations in two different materials, corresponding to structures and experimental conditions used in Ref. [28] (**a**) and Ref. [15] (**b**). Color legend refers to the potential throughout the structure under negative biasing. Detailed overview of the layer stack in the boundary region between the oxide and the 2D material layer is as follows: (**a**) structure for simulating the oxidation of graphene in a domain with extension up to 30 µm, using a central 6 µm radius tip biased at −30 V with respect to ground, which is located at the periphery of graphene, and all above an insulating quartz substrate of 90 nm. (**b**) Structure for simulating the oxidation of a 20 nm thick MoSe_2_ flake on a gold substrate (note bottom surface grounded) in a domain extending up to 60 nm. According to [15], a 10 nm radius tip is biased at −27.5 V with respect to a ground surrounding the flake to resemble the contact with the gold substrate.

To determine the electrical magnitudes around the boundary between the oxidized and non-oxidized layers (charge density electric field components and potential), we propose a more complex and realistic framework including a multilayer stack of the different materials involved in the process as follows: oxide, 2D semiconductor layer, wetting layer, insulating substrate, tip fingerprint biased at a certain voltage V_b_, and metal contacts, as those shown in Figure 1. For effective management of such a complex system, we use a continuous layer approach. While a continuum-based model may potentially lose features associated with the discrete nature of 2D materials, it is unique to reveal the electrical magnitudes across a complex multilayer with realistic dimensions and provide qualitative insight into the main causes driving the process. For that purpose, we solve the Poisson equation with boundary conditions using finite element calculations along the whole structure.

This assumption has been validated by comparing the results obtained by this approach with those derived theoretically on certain test structures involving pure 2D sheets with zero thickness (see Section S1 of Supplementary Materials) [35,36]. Using this tool, we will demonstrate the ability to assess, for example, the energy barrier for incorporating oxyanions in the presence of an electric field, as well as its evolution as the oxide expands. Hereinafter, we will particularize the layer stack to those samples including graphene (displayed in Figure 1a) and to those including MoSe_2_ flakes which are at the nanoscale (Figure 1b). In both cases we have employed multilayer structures and experimental conditions (tip radius, applied voltage, etc.) that align with those utilized in two experimental references as follows: [28] for graphene and [15] for MoSe_2_. These cases are used to verify the validity of the model and are discussed below.

The most relevant characteristics of the semiconductor layers included in the models are collected in Table 2. The thickness attributed to a graphene monolayer transferred onto a certain substrate is typically higher than that of the monolayer in vacuum, depending on the conditions of the substrate surface and adsorbates. Atomic force microscopy (AFM) yields a variety of results in the range from 0.4 nm to 1.7 nm when measuring the monolayer graphene thickness on a substrate, because of tip-surface interactions and the influence of imaging forces [37,38]. In this work, for simplicity, we have rounded the graphene thickness to 1 nm. Since graphene is a highly conductive layer, variations in a few angstroms around this thickness do not significantly alter the subsequent electrostatic results of the work. In the case of MoSe_2_ flakes of Ref. [15], where a number of monolayers are involved, we have considered, for simplicity, that the stack behaves as a homogeneous semiconductor with the monolayer properties and the reported thickness of 20 nm. Regarding the thickness of graphene oxide (GO) functionalized directly on graphene, our own measurements using AFM indicate that it raises about 1 nanometer above the graphene surface [28]. Other studies report a 1 nm step over a mica substrate for GO flakes [39], although it has been confirmed that the GO thickness depends on the degree of oxidation [40]. In this work, we have included our own determination, as it aligns precisely with the outlined structure, assigning a thickness of 2 nm to GO. Regarding the thickness estimation for the oxidized MoSe_2_, which is considered a non-stoichiometric compound (MoO_x_+SeO_2_), there is currently a lack of data. Given that the theoretical thickness of MoSe_2_ is approximately twice that of graphene [41,42] and in view of the measured step at the initial stages of the oxide growth [15], we considered an oxide thickness of 3 nm for simplicity.

Regarding the relative dielectric permittivity of GO, the reported measured values span a range from 10^4^ to almost 10^6^ [45,46]. This is attributed to the giant orientational polarizability of oxygenated bonds. To that end, we have conducted simulations with a scope of values from 10^2^ to 10^5^. In the case of the oxidation of MoSe_2_ in ambient conditions, a qualitative evolution with time from large polar oxygen bonds to non-polar oxide has been reported along this process [50]. In light of this, simulations with permittivity from 20 (that corresponding to MoO_x_) to 10^4^ have been performed. The latter values would correspond to the initial polar bonds mentioned in [50] and could tentatively resemble those obtained in graphene.

Further details such as structure, symmetry, and working conditions are described in Section S2 of Supplementary Materials. To solve the conventional Poisson equation with boundary conditions by finite element calculations, commercial software has been used [20]. Unlike previous electrostatic approaches which treated 2D layers as either dielectric or pure conductors, the used software allows 2D layers to be considered as doped semiconductors and therefore calculates self-consistently the equilibrium space charge distribution, electric field, and potential at any given point. The remaining layers—oxidized 2D material, substrate, and wetting layer—are considered pure dielectrics for simplicity.

### 3.2. Electrostatic Results Around the Boundary Between Oxidized and Pristine Regions in Graphene

**Figure 2:**
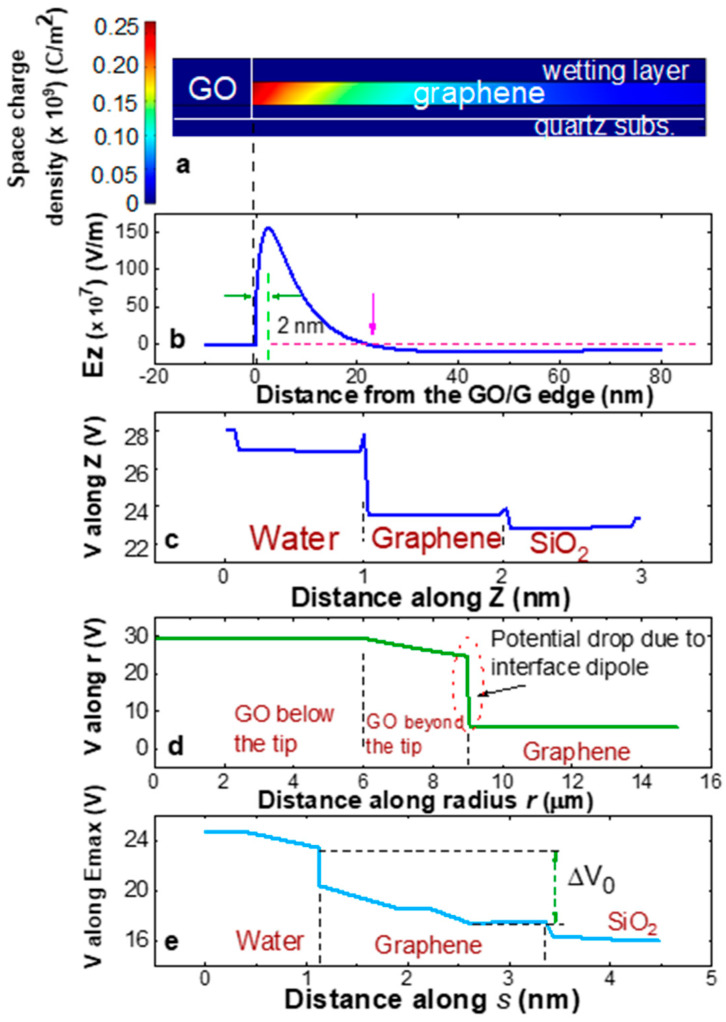
Electrical magnitudes plotted inside graphene (G), calculated from the structure of Figure 1a near the GO/graphene interface located at r_GO_ = 9 µm from the tip axis, considered as the origin (r = 0 nm in the plot) for negative biased probe at V_b_ = −30 V. (**a**) Space charge density (C/m^3^). (**b**) Vertical component of the electric field, Ez, along a radius beyond the GO/graphene boundary. (**c**) Negative potential along a vertical path (Z) inside graphene at 2 nm from the GO/graphene boundary. (**d**) Potential along a radius. (**e**) Potential along the direction of the maximum electric field ($\vec{s}$) at 2 nm from the GO/graphene boundary.

The ability of this tool to elucidate the dynamics of oxide propagation in graphene is substantiated by Figure 2, where the distribution of relevant magnitudes such as the space charge density (Figure 2a), vertical electric field component (Ez) (Figure 2b), and the electrostatic potential along different directions (Figure 2c–e), have been plotted in the region close to the boundary between GO and graphene in the structure of Figure 1a, with the probe negatively biased with respect to the grounded graphene.

As illustrated in Figure 2a, when a negative bias is applied at the probe, a huge dipole is produced at the boundary GO/graphene. This is because the polarization charge at the edge of the dielectric GO induces a mirror-positive free charge in the nearby p-type graphene. The band misalignment and difference in bandgap between GO and graphene prevents diffusion of the graphene holes towards the insulating layer. Then, the combination of a high polarizability in the insulator oxide with abundant free charge in the adjoining layer results in a significant upturn or sharp increase in the electric field within the semiconducting layer (Figure 2b). Commercial graphene shows up as a particularly suitable material for this effect due to its residual p-type doping of approximately 5·10^12^ cm^−2^ [44]. Figure 2b illustrates that the electric field comes from being very low in oxide due to dielectric screening, then exhibits a pronounced intensity peak (>10^9^ V/m) within graphene, at approximately 2 nm from the boundary, and finally decays rapidly with *r* from that maximum.

**Figure 3:**
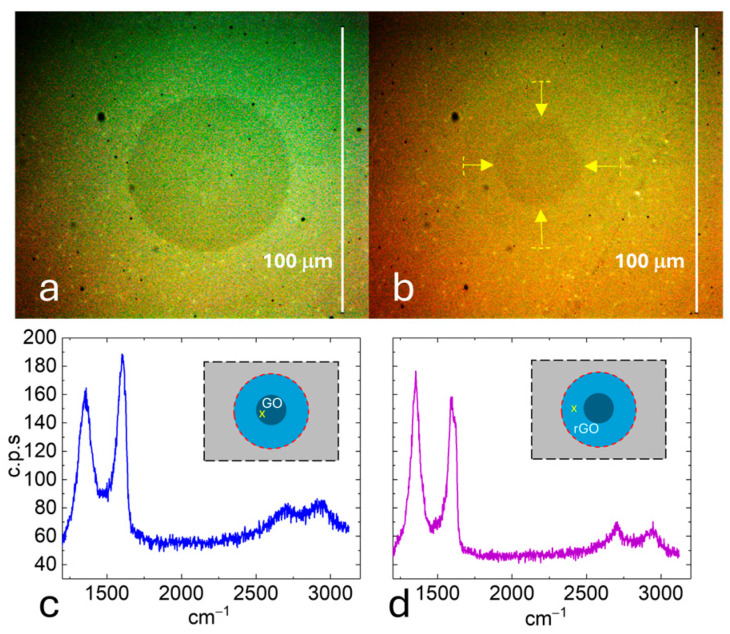
(**a**) Circular spot of graphene oxide obtained at bias voltage V_b_ = −30 V and RH = 92% (T = 20 °C). (**b**) Partially reduced spot resulting from a sharp voltage change to +50 V. The cathodic reduction takes place from the outside to the inside, as indicated by the circular crown with soft contrast (yellow arrows zone). (**c**) Raman spectrum (blue line) recorded from the central, oxidized region of the reduced spot in (**b**), as marked in the inset. (**d**) Raman spectrum (purple line) recorded from the region reduced after oxidation in the spot (**b**), as marked in the inset.

In the presence of a wetting layer resulting from a sufficient degree of RH (>70%), the orientation of this field is conducive to accelerating oxyanions (OH-) towards graphene, thereby facilitating their bonding. Note that the region where OH- incorporation to graphene is favored (for Ez > 0) does not extend indefinitely but is limited to a narrow zone of about 30 nm near GO, until the pink arrow marked in Figure 2b. This effect is considered as responsible for the sharp contrast between oxide and pristine graphene observed by microscopy. Furthermore, it contributes to the regular, circular shape of the oxide spot as it expands [28] (see Figure 3a). The dipolar electric field acts as a driving force for the process by inducing a drop in the potential function (ΔV_0_) from the wetting layer to graphene. This is illustrated in Figure 2c–e, which show the potential along the following three different paths: vertically, V(z) (Figure 2c); horizontally in the radius direction, V(r) (Figure 2d); and in the direction $\vec{s}$ of the maximum electric field vector, V(s). Since V(z) and V(s) are functions that vary along the horizontal radius, they are plotted at the point of maximum Ez field (2 nm near the boundary GO/graphene).

An interesting experimental confirmation that the oxidation/reduction process is conducted by the dipolar field at the perimeter of the oxidized region is shown in Figure 3. In the experiment, a conventional GO spot performed at V_b_ = −30 V and RH = 92% (T = 20 °C) in a graphene monolayer on quartz substrate is shown in Figure 3a (circle of dark contrast). Then, when the voltage is changed from negative to positive immediately after oxidation (V_b_ = +50 V), the chemical reduction in the previously oxidized spot mainly occurs, counter-intuitively, from the outer edge to the center (see the soft contrast left by the reduced zone in Figure 3b) and not from the inner region of the spot close the probe (which still shows the darker contrast associated with a highest degree of oxidation). This is confirmed by Raman spectroscopy, where spectrum of Figure 3c was recorded from the central, oxidized region of spot 3b (marked in the inset), and spectrum of Figure 3d was recorded from the reduced region of the same spot (see the inset). With respect to the degree of reduction achieved, the width and relative intensity of the recorded Raman modes in the reduced region are equivalent to those obtained from GO flakes after more than 2 h under hydrazine vapor or, alternatively, after annealing at a minimum of 1000 °C [51].

### 3.3. Formulation of the Expansion of the Oxidized Region over Time in Graphene

A preliminary formulation for the expansion of the oxide radius over time was previously proposed [28]. In that work, the probability that one oxyanion incorporates covalently to graphene, P_1OH_, is considered to obey the classical Boltzmann statistics, and depends essentially on the energetic barrier to break the 2D layer bonds. Below we present a detailed formulation derived from fundamental principles, which ultimately yields an expression for the increase in the radius of an oxide spot as a function of time.

After identifying the driving force behind the incorporation of oxyanions around oxidized areas by applying a local electric field, the formulation begins establishing the differential relationship between the attachment of oxyanions (OH-) and the resulting increase in the spot radius (r_GO_).

(1) $$d\left({OH}^{-}\right)=\frac{2\pi r_{GO}}{S_{OH}}dr_{GO}$$

where S_OH_ represents the effective area of one oxyanion. S_OH_ may be estimated from the degree of oxidation typically obtained by the different techniques. In case of field-assisted oxidation, a typical O/C is 0.35 [28]. Chemical techniques can achieve O/C ratios as high as 0.8 by applying Hummers’ methods [52], though this approach is not applicable in this case.

The probability that one oxyanion close to the boundary covalently attaches to the material, P_1OH_, depends on the aforementioned energetic barrier (W_0_). This probability can be formulated according to the classical Boltzmann statistics. In this scenario, the dipolar field at the interface between the oxide and pristine graphene is expected to produce a reduction in the energetic barrier ΔW_0_ (as illustrated in Figure 2). This reduction is, in turn, dependent on the radius r_GO_. Then, under an external voltage, P_1OH_, is expressed as:

(2) $$P_{1OH}=K\cdot e^{-\frac{\left(W_{0}-∆W_{0}\left(r_{b}\right)\right)}{kT}}=\tilde{K}{\cdot e}^{\frac{∆W_{0}(r_{GO})}{kT}}$$

where $\tilde{K}=K\cdot e^{-\frac{W_{0}}{kT}}$ is a parameter in [s^−1^].

The total rate of incorporation of oxyanions, d(OH^−^)/dt, can then be described by the probability of attaching one oxyanion (P_1OH_) multiplied by the number of available sites (C atoms in the case of graphene) along the entire spot perimeter (N_OH_), as well as the probability of one site being occupied (P_S_) in terms of density of present oxyanions around. It is at this factor that the role of relative humidity must be considered:

(3) $$\frac{d({OH}^{-})}{dt}=P_{1OH}\cdot (N_{OH}\cdot P_{S})=P_{1OH}\cdot (2\pi r_{GO}\cdot f\left(RH\right))$$

where the product N_OH_·P_S_ has been expressed as the perimeter of the spot multiplied by a certain function f(RH) that ultimately depends on the relative humidity.

By substituting (2) in (3),

(4) $$\frac{d(OH)}{dt}=2\pi r_{0}f(RH){\overset{ˇ}{r}}_{GO}\tilde{K}e^{\frac{∆W_{0}(\overset{ˇ}{r})}{kT}}$$

where r_0_ is the probe radius, and ${\overset{ˇ}{r}}_{GO}=\frac{r_{GO}}{r_{0}}$ is the dimensionless relative radius of the oxide in relation to that of the probe.

**Figure 4:**
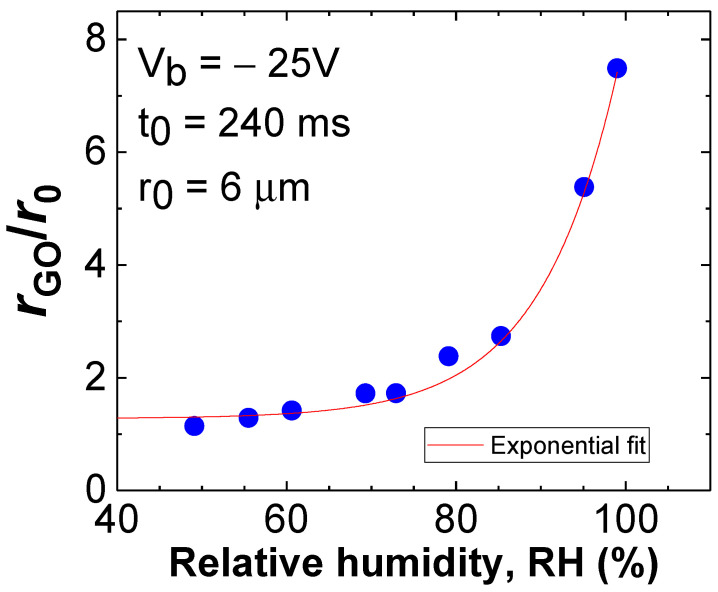
Evolution of the GO radius with respect to that of the tip versus relative humidity (RH) for fixed V_b_ = −25 V and exposure time t_0_ = 240 ms. Solid red line represents an exponential fit to the experimental points.

Taking advantage of the relation between (OH) and *r_GO_* in (1), we can finally write the expansion speed of the relative oxide radius, ${\overset{ˇ}{dr}}_{GO}$/dt, as:

(5) $$\frac{d{\overset{ˇ}{r}}_{GO}}{dt}=\frac{1}{\alpha_{0}}e^{\frac{∆W_{0}(\overset{ˇ}{r})}{kT}}$$

where $\alpha_{0}=\frac{r_{0}}{A_{OH}\cdot f\left(RH\right)\cdot \tilde{K}}$ [s]. From which, by integration, a function *t*(ř) describing inversely the expansion of the relative radius along time is obtained.

(6) $$t=\alpha_{0}\int_{1}^{{\overset{ˇ}{r}}_{GO}}e^{\frac{e(W_{0}-∆W_{0}({\overset{ˇ}{r}}_{GO}))}{KT}}d{\overset{ˇ}{r}}_{GO}$$

where α_0_ is a scale parameter that includes, among other factors, the dependence on the relative humidity RH. It is important to note that when fixing experimentally a certain exposure time and a bias voltage, the increase in the oxide relative radius with the RH results in a nonlinear function. Figure 4 illustrates the evolution of the relative GO radius versus RH for V_b_ = −25 V, and exposure time t_0_ = 240 ms. Based on empirical evidence and within the margin of error, it can be considered to follow an exponential trend (solid line in Figure 4).

In Equation (6), ΔW_0_ represents the variation in the energy barrier for oxyanion incorporation at the edge GO/graphene due to the influence of the dipolar electric field. This is the key function that explains the enhancement of the oxidation rate with the field, and its dependence on the distance to the tip. For any applied voltage, the following three critical factors influence on ΔW_0_: (i) the radius r_GO_ with respect to that of the tip (r_0_) and to layer thickness (*d*); (ii) the oxide polarizability, considered in our simulations as static relative permittivity, ε_r_; and (iii) conductance of the 2D material (involving type and level of doping and carrier mobility). The layer scheme proposed in Figure 1 allows tracking the influence of each one by applying finite element calculations throughout the entire structure.

For that purpose, we have used the electrical characteristics of graphene described in Table 2 [42,43,44,45,46]. The energy function ΔW_0_(r) has not been described in the literature. A preliminary and rough analytical expression was assumed in Ref. [28]:

(7) $$\Delta W_{0}\;=\;e\cdot E_{r}({\overset{ˇ}{r}}_{GO})\Delta r_{p}$$

where *e* is the elementary charge, E_r_(${\overset{ˇ}{r}}_{GO}$) is the radial component of the electric field at the boundary GO/graphene, and Δr_p_, was left as a fitting parameter, subsequently obtained of a few nm.

In this work, we look for a more reliable determination of the function ΔW_0_(r) and its dependency on the previous factors. To that end, we have developed simulations of electrostatic properties on multilayers of Figure 1, which resemble real samples. In this framework based on continuous layers, our hypothesis is to assume that ΔW_0_ is represented by the drop ΔV_0_ in the potential V(s) from the wetting layer to the 2D material, along the direction of the maximum electric field (Figure 2e). For any GO radius, this potential drop is evaluated close to the boundary GO/graphene, at the point of Ez maximum (plotted in Figure 2c,e). The aim of this work is to demonstrate the usefulness of this approach.

**Figure 5:**
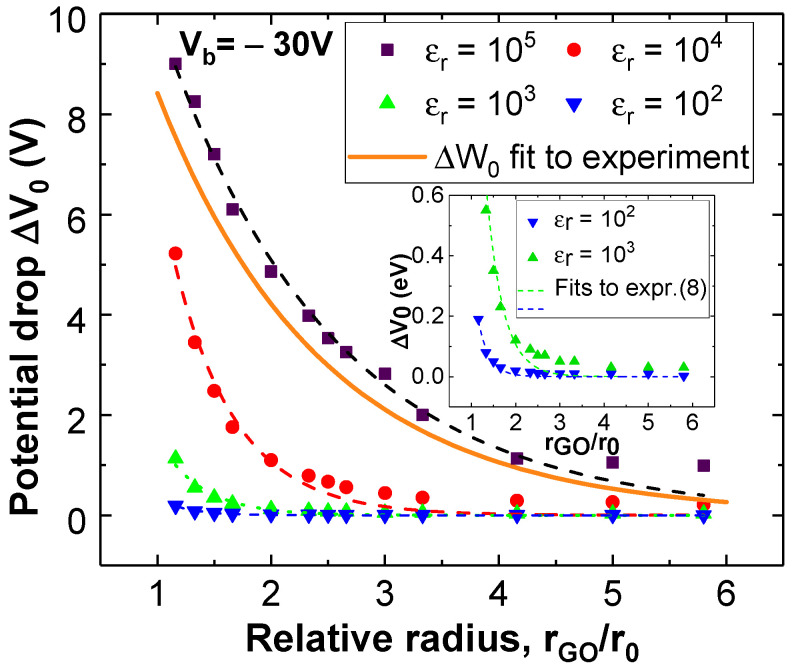
Potential drop (∆V_0_) from water to graphene obtained by a numerical simulation and evaluated according to the trends in Figure 2e, against a different GO relative radius, considering a 6 μm radius tip (r_0_). The permittivity ranges from 10^2^ ≤ ε_r_ ≤ 10^5^. Dashed lines are least square fits of these values to expression (8). For clarity, inset shows zoomed plots for permittivity values of 10^2^ and 10^3^. Solid line represents ∆W_0_ as obtained from fitting (6) to the experimental data.

In Figure 5, the drop ∆V_0_ from the wetting layer to graphene, as depicted in Figure 2e, was evaluated and plotted against GO relative radii (${\overset{ˇ}{r}}_{GO}$) in the range 1.33–5.83. The GO permittivity is also varied in the range 10^2^ ≤ ε_r_ ≤ 10^5^. In view of the tendency observed, maximum when ${\overset{ˇ}{r}}_{GO}$ → 1 and null for ${\overset{ˇ}{r}}_{GO}$ → ∞, a decaying exponential fit shows up as a good empirical approach for the evolution of the voltage drop with ${\overset{ˇ}{r}}_{GO}$. Thus, the expression for ∆V_0_ can be defined by an exponential form:

(8) $$∆V_{0}({\overset{ˇ}{r}}_{GO},V_{b})=\beta_{0}V_{b}e^{-\gamma_{0}({\overset{ˇ}{r}}_{GO}-1)}$$

where V_b_ is the applied external voltage, and β_0_ and γ_0_ are parameters to be obtained by least-square fitting, as explained below. Note that the ratio of the tip radius to the active layer thickness, r_0_/d, is included within the γ_0_ parameter in concordance with [36], and thus accounts for the scale changes in the tip diameter.

According to the hypothesis that *e*·∆V_0_ conceptually represents a similar magnitude to ∆W_0_, an equivalent form to (8) will be adopted for the barrier reduction ΔW_0_(${\overset{ˇ}{r}}_{GO}$) shown in Equation (6).

**Figure 6:**
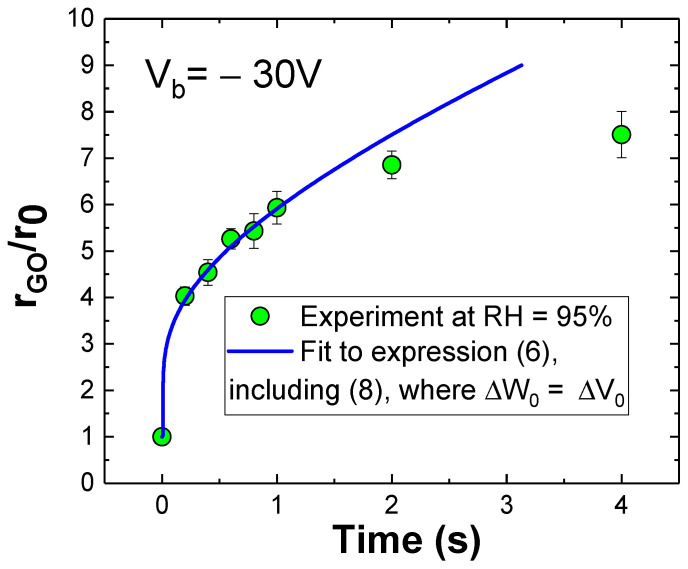
Evolution of the relative radius (r_GO_/r_0_) of the graphene oxide spots as the contact time between tip and graphene (exposure time) is prolonged. Operative voltage is V_b_ = − 30 V and relative humidity RH = 95%. Error bars result from the average of 12 spots. Solid line is the fit to the oxide growth model in (6), including expression (8).

Thus, function (8) is introduced in (6) to complete a model with three fitting parameters (α_0_, β_0_, and γ_0_). Next, the measured experimental evolution of the spot relative radius as a function of time [28] can be fitted to the completed expression (6). The result is shown in Figure 6 as the blue solid line, where V_b_ = −30 V and relative humidity RH = 95% have been considered.

The observed deviation in the spot behavior beyond two seconds, as compared to the model’s prediction, is associated with the deformation of the spots’ circular shape. At this limit, for large spot diameters (r_GO_/r_0_ > 6), it is interpreted that the dipolar electric field driving the expansion is comparable to the fluctuations caused by lattice defects (grain boundaries, dust particles, etc.). This results in irregular progress of oxidation.

The resulting ∆W_0_ from the model’s fitting to the experimental data is represented by the orange solid line in Figure 5. Interestingly, within the existing uncertainty for the value of the electrical permittivity of graphene oxide (close to ε_r_ = 10^5^) [45,46], there is a good agreement between the ∆W_0_ obtained by fitting *t*(${\overset{ˇ}{r}}_{GO}$) to the experimental data, and the energy drop *e*ΔV_0_ solely derived by finite element simulations using the simple framework proposed in Figure 1a. In conclusion, the straightforward structures and methodology outlined above provide a fairly reliable approach to predict the progression of graphene’s field-assisted local oxidation, based solely on intrinsic material properties. In the next subsection we apply these trends to the oxidation of thin films for a transition metal dichalcogenide such as MoSe_2_, whose oxidation dependence on time has been reported in a previous work [15].

### 3.4. Expansion of the Oxidized Region over Time in Transition Metal Dichalcogenide 2D Layers at the Nanoscale

In order to apply the previous framework to some TMD layers one has to take into account the following three main differences with respect to our experiments in graphene: (i) oxidation of these materials in ambient atmosphere gradually evolves from both hydroxyl species and surface dichalcogenide moieties X^2−^ (X being S, Se, etc.) to more stable oxo-bridged dimolybdenyl Mo(V, VI) species and acids [50]. It means that at the initial stages of the process the oxidized species may exhibit high orientational polarizability due to the incorporation of OH-, but this value should eventually converge on that of the MoO_3_, which has a much lower ε_r_, of around 18–20. Currently, the evolution of this process (species and time) is not yet characterized for LAO, where the electric field can affect substantially the oxidation rate. (ii) Many TMD layers grown by MOCVD are wide bandgaps with n-type residual doping [48,49,53]. This contrasts with the usual quasi-null gap and p-type doping of as-grown graphene [42,44]. As will be shown, n-type doping does not prevent oxidation but does reduce the rate at which it occurs. (iii) The available data are from nanoscale experiments, so the simulations must be scaled accordingly.

**Figure 7:**
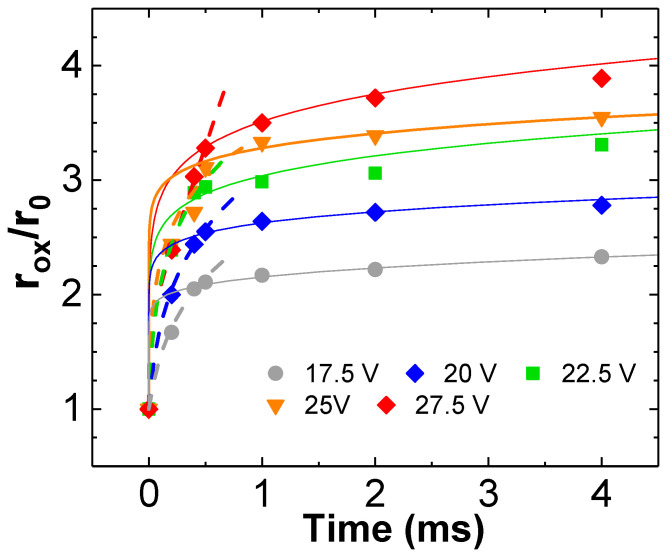
Experimental points from Ref. [15], where the evolution of the relative radius for the MoSe_2_ oxide (r_ox_/r_0_) is measured as the contact time between tip and sample is prolonged. Operative voltage varied in the range −17.5 V ≤ V_b_ ≤ −27.5 V. A tip with radius r_0_ ≤ 10 nm was used, and the experiments were performed at a relative humidity of 60–65%. Solid lines represent the fit of the whole set of data to model (2) for each V_b_, whereas dashed lines represent the fits up to exposure time t < 1 ms.

The evolution of the oxide spots in MoSe_2_ platelets (around 20 nm thick) performed with a scanning probe nano-scope of tip radius r_0_ ≤ 10 nm, along an exposure time up to 4 ms and for different bias voltages (from 17.5 V to 27.5 V), is shown in Figure 7, according to Ref. [15]. Two trends can be observed in all plots: (i) a rapid increase in the relative radius at the initial interval, up to approximately t ≤ 1 ms and (ii) subsequent growth tends to saturate from t > 1 ms. Solid lines, *t*(${\overset{ˇ}{r}}_{OX}$), are the fits using expressions (6) and (8) to the whole data collection corresponding with each operative voltage. It can be observed that due to the saturation behavior, our physical model struggles to fit the entire set of experimental data for any applied voltage. It should be noted that those experiments were conducted at intermediate RH values of approximately 60–65%. Since TMDs are generally considered hydrophobic [54,55], it is expected that these conditions will result in the formation of a partial or insufficient wetting layer, thereby leading to oxidation eventually limited by oxyanions supply, i.e., dominated by the water meniscus below the tip. It appears that the water bridge extends from 2 to almost 4 times the tip radius, depending on the working voltage. However, the available data permit the application of the above-described physical principles only to the oxide expansion in the interval prior to saturation. The dashed lines in Figure 7 represent the fits of *t*(${\overset{ˇ}{r}}_{ox}$) to those data with exposure time up to t < 1 ms, at the corresponding V_b_. This way, a new function ΔW_0_(${\overset{ˇ}{r}}_{ox}$) is obtained for each fit.

**Figure 8:**
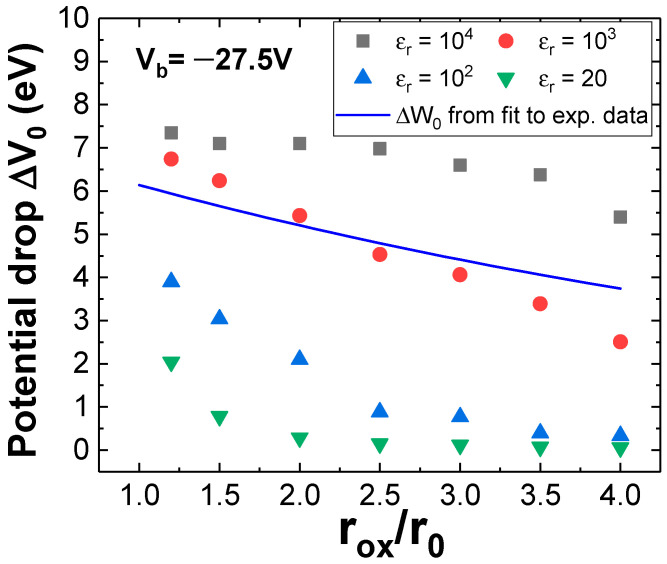
Numerically calculated potential drop ΔV_0_ from the adsorbed wetting layer to MoSe_2_, induced by an applied external voltage V_b_ = −27.5 V, at various relative radius (r_ox_/r_0_, where r_0_ = 10 nm). ΔV_0_ is obtained at the Ez maximum, approximately 2 nm beyond the boundary MoO_x_/MoSe_2_, and from the interface to a depth of 2 nm in the 2D flake. Solid blue line represents ∆W_0_ as obtained from fitting (6) to the corresponding experimental data [15].

The resulting ΔW_0_(${\overset{ˇ}{r}}_{ox}$) for V_b_ = −27.5 V is represented by the blue solid line in Figure 8. In addition, *e*ΔV_0_ is obtained by finite element calculations on the layer structure displayed in Figure 1b for V_b_ = −27.5V, including variation in the oxide permittivity from 20 to 10^4^ (dots of Figure 8). As with graphene, the comparison of *e·*ΔV_0_ with ΔW_0_ in Figure 8 indicates that the numerical simulation based solely on the material properties is consistent with the model fitted to the experimental results for certain oxide permittivity, close to ε_r_ ≈ 10^3^. This is in line with the mentioned existence of high polar species reported at the initial state of oxidation [50]. Even in case of high humidity conditions (RH > 90%) with a well-developed wetting layer, simulations of the electric field in proximity to the oxide edge suggest that a decline in permittivity due to the evolution of the oxide species (as outlined in [50]) would result in a substantial reduction and broadening of the vertical electric field (Ez) values, eventually leading to negative Ez values at the boundary, thereby halting the oxidation process.

This phenomenon is attributed to insufficient screening of the negative polarization charge at the oxide perimeter by the n-type material, thereby inducing a rapid saturation of oxide growth (see Section S3 of Supplementary Materials). In this case, the n-type character of the doping is decisive, since if the doping were p-type, as in Si, the incorporation of oxyanions would always be favored, and the aforementioned saturation would hardly be noticeable. In fact, such abrupt saturation is not observed in LAO experiments performed on Si, even up to spot radii hundreds nm long [11].

## 4. Discussion

Local anodic oxidation is a noteworthy technique for the functionalization of materials. Advantages of this process include its cleanliness, the robustness attributed to covalent bonds, and its precise positional control. However, this process can result in a loss of material’s conductive properties. This may be a critical aspect in the field of biosensors that measure electrical characteristics, such as those based on field-effect transistors (FETs). Consequently, the employment of a predictive tool to adjust the size of the oxidized points according to the dimensions of the FET channel is of significant utility.

Due to the simplicity of the proposed model, which works with continuous and isotropic materials, no particular considerations have been made regarding the properties of the material surfaces. However, it is necessary to make some clarifications regarding the relationship between the model parameters and the experimental complexity of the oxidation process of 2D materials.

The material’s hydrophobic/hydrophilic character, influenced by the underlying substrate (a phenomenon referred to as wetting transparency), plays a pivotal role in the oxidation progress [56,57]. In addition, the adsorption of water or other chemical compounds present in the environment or during the processing of the material, point defects and grain boundaries, or the anisotropy of the atomic structure (typical of materials such as TMDs) are also relevant aspects in the oxidation process.

However, given the general way in which the factors involved in Equations (3)–(5) are defined, we can conclude that the effects of several of the aforementioned aspects are quantitatively subsumed in these factors. In Equation (3), the only factor that depends on the experimental conditions is Ps, which expresses in a standard way the probability of occupying a site as a function of the number of available oxyanions. Note that the factors P_1OH_· and N_OH_· are not directly dependent on the experimental conditions and can be expressed as indicated in Section 3.3. In this context, the factor Ps encompasses all the effects that influence the oxyanions supply as follows: adsorbed water molecules that may be present in the wetting layer, material’s hydrophobicity, or even oxygen radicals coming from partially dissolved material oxides as observed in TMDs [58]. The relationship between Ps and these experimental features is challenging to analyze. This is why we express it as a general function of relative humidity through f(RH) in Equation (3). As a result, the oxide radius increases in a nonlinear way with RH (Figure 4).

Conversely, the electrostatic multilayer model depicted in Figure 1 involves a continuous wetting layer with permittivity ε_r_ = 80 (refer to Section S2 of the Supplementary Materials), which covers the entire 2D material. It is important to note that this does not impose Ps = 1, as partial or insufficient coverage of the wetting layer can be considered an average reduction in the permittivity of this layer, with ε_r_ ranging from 80 (total coverage) to 1 (air, no coverage). The results of Figure 5 have been obtained for a continuous wetting layer with ε_r_ = 80, which in this context is equivalent to Ps = 1. In coherence, the experimental data that generated the continuous curve in Figure 5 were obtained for a RH of 95%, which is close to the maximum.

The number of single layers involved in the material are not parameters directly included in the general formulation described in Section 3, but their influence is manifested through the modifications they can cause in the electric field, and eventually in the reduction in the energy barrier, ΔW_0_, for the incorporation of oxyanions. For instance, the MoSe_2_ flakes referenced in [15], to which the model is applied, are deposited on a gold metal substrate that is grounded with respect to the probe voltage. The number of layers contribute to the proximity of the ground plane to the probe, thereby influencing the electric field. In the case of graphene, given its semi-metallic character and the fact that the graphene layer is grounded at the sample’s periphery, adding a second layer below the one to be oxidized must significantly impact the electric field. This structure is similar to locating a ground plane just below the layer to be oxidized. Therefore, the progression of oxidation should undergo significant changes with respect to those of Ref. [28]. We are currently obtaining preliminary experimental results on bilayer graphene that confirm this hypothesis. The application of the proposed model to these samples is left as future work. In most structures, the field distribution is not readily obtained analytically, underscoring the importance of finite element calculations on a particular structure, such as those in Figure 1.

With regard to the anisotropy exhibited by the lattice of some TMDs, the model proposed here is isotropic and does not consider dielectric properties in the form of an anisotropic tensor for the structures in Figure 1. In particular, Ref. [15] presents an example in which, under conditions of low relative humidity (RH < 50%) and at the initial stages of the process, oxidation initiates in a triangular shape, inheriting the anisotropy of the crustal lattice. However, when RH increases (RH > 60%) and, therefore, both the water meniscus and the electric field lines spread, then the triangular anisotropy is diluted, and the shape of the spot tends to become circular. This experiment unveils the transition between two distinct oxidation regimes as follows: when RH is low, the scale becomes smaller and the oxidation is limited by the oxyanion supply at the water meniscus. At this scale, oxidation may exhibit anisotropy according to the lattice structure. Alternatively, if RH is sufficiently high to guarantee oxyanion supply up to longer distances, the local anodic oxidation process tends to be dominated by the electrostatic dipolar field at the boundary between oxidized and non-oxidized regions, and the anisotropy is gradually lost with distance from the point where the potential is applied. This is an indication that reinforces the principles presented in this model. The calculations developed in Figure 6 and Figure 7 have been applied to these latter experimental conditions of Ref. [15]. This topic was previously discussed in Section 3.4.

In the structures depicted in Figure 1, all layers, with the exception of the 2D material (graphene and MoSe_2_), have been modeled as pure dielectrics. In the specific instance of GO, this is an approximation. Nevertheless, the substantial disparity in the conductive properties of graphene and its oxide renders the assumption a highly accurate estimation, thereby significantly reducing computation time (which typically spans several hours for each of the simulated cases) without altering the conclusions of the study. The incorporation of semiconductor properties to the oxidized materials will be a subject to be addressed in future research endeavors.

Other effects that have not been taken into account in our model are the possibility of tunnel current from the free holes in graphene to GO. The band alignment at the interface between GO and graphene is presumably type I, given the quasi-zero gap of graphene. However, the higher work function of graphene oxide tends to shift this alignment towards type II [59]. In any case, this results in a barrier for the free holes recombination (which precisely holds the bipolar action). This barrier could be overcome by the tunnel effect, eventually including an additional current component. However, it is important to note that within our proposal, components that increase the parallel current, such as Mott–Gurney [28] and tunnel current, should contribute to reducing the electric field. Consequently, the tunnel effect would eventually limit the oxidation rate.

On the other hand, photon-assisted local anodic functionalization is an emerging field with significant potential. In general, it would be expected that any Floquet state contributing to tunneling will slow down the oxide progress. However, the potential impact of light application on the electronic excitation of GO through electron–photon entangled states should be considered in future works, as it may contribute to enhancing the dipolar nature of the GO–graphene interface and eventually the oxidation progress.

Finally, the insulating GO spot surrounded by a cloud of free holes concentrated in the near 30 nm displays a specific architecture that may be considered the basis of a topological insulator, since it is an insulator inside but highly conductive at the perimeter. From this point on, interaction with light of a specific frequency can create Floquet states in this interface. These states change the conduction properties, opening up a wide range of possibilities in Electronics.

## 5. Conclusions

In summary, a model grounded in physical principles has been developed to describe the phenomenon of electric field-assisted local functionalization in 2D materials. The model illustrates how the oxidized spot’s radius expands horizontally over time, driven by the dipolar electric field at the boundary between oxidized and pristine material, which results from the applied voltage. To that end, Boltzmann statistics and relevant physical quantities are taken into account, including the energy barrier for the incorporation of oxyanions and its dependence on radius. This regime is particularly effective in conditions that ensure a substantial wetting layer, facilitating the supply of oxyanions from the immediate environment. According to the materials under consideration (silicon, graphene, and MoSe_2_ flakes), this typically takes place when the relative humidity (RH) is greater than 60–70%.

This approach addresses an area that was not covered by the previous water meniscus paradigm, in which the description of the lateral expansion of the oxidation was dominated by empirical models. Furthermore, the model elucidates the pronounced contrast observed between oxidized and non-oxidized regions. This contrast can be attributed to a substantial increase in the electric field intensity that occurs exclusively at the boundary between those regions.

The as yet unknown energy barrier for the incorporation of oxyanions and its dependence on radius has been determined by adjusting the model to experimental results, using both data from this author’s own research (for graphene) and data from other authors in the literature (for TMDs). This attribute confers upon the model the capacity for predictive functionality under a range of operating conditions, encompassing applied voltage, exposure time, probe radius, and temperature.

Furthermore, an electrostatic model based on thin multilayers was proposed; it allows the prediction of oxidation behavior based solely on the intrinsic properties of the material. This model incorporates the doped semiconductor characteristics of the layer of the 2D material to be oxidized.

Alternatively, by measuring the evolution of the oxidized spot over time, the model can be used to estimate material properties, such as the effective dielectric constant of the oxide. The results obtained for GO and the precursor species that initially lead the oxidation of MoSe_2_ are approximately 10^5^ and 10^3^, respectively.

The employment of finite element simulations on a fundamental multilayer structure incorporating 2D materials facilitates the anticipation of the expansion of the oxidation radius over time. This development signifies a substantial advancement in the domain of biosensor fabrication, as it enables precise estimation of the dimensions of functionalized domains. This is a pivotal step preceding bioreceptor attachment. Its predictive nature is of great interest for establishing the operating conditions necessary to functionalize large regions (in the range of tens of µm) controlled at high speed within the channel of FETs for biosensors.

Eventually, the model contributes to a comprehensive explanation of the rapid saturation of oxide size in TMDs during LAO at nanoscale, as seen at intermediate relative humidity values. This phenomenon can be attributed to a combination of the following three factors: the extension of the water meniscus, the n-type character of the material, and the potential evolution of the oxide from a highly polar species to the significantly lower polarizability of the MoO_3_ compound.

## Figures and Tables

**Table 1:** Characteristics of empirical approaches used to fit the evolution of oxidation in the lateral direction to the experimental data. w(t) represents the diameter function over time, and V is voltage.

Authors and Refs.	Expression
Dubois et al. [8]	w(t, V) = V/V_0_ (t/t_0_)^α^ (“power-of-time law”) parameters V_0_, t_0_ and α
Calleja et al. [26]	w(t,V) = w_0_(V) + w_1_(V) ln t (“direct-log form”) w0 and w1, linearly dependent on V
Dagata et al. [25]	w (t,V) = 2 r_0_ + 3 V ln(t/τ + 1) + 1.5V^2^ t^0.5^, parameters r_0_ and τ
Filipovic et al. [24]	w(t,V) = w_0_(V) + w_1_(V) (ln t)·f(RH) w0 and w1 linearly dependent on V, and f(RH) linearly dependent on relative humidity (RH)
Borodin et al. [15]	$w=\;\cdot ln{\left(R\cdot D_{eff}\frac{t}{t_{0}}+1\right)}^{\alpha }$ Parameters D_0_, R, D_eff_, t_0_, α

**Table 2:** Characteristics of Materials Used in Physical Simulations.

	Thin Film Thickness (nm)	Oxide Thickness (nm)	Band Gap (eV)	Doping in the Model (cm ^− 3^)	Range of Oxide Relative Permittivity Used
**Graphene**	1	2	0.020 ^a^	[p] = 10^20 b^	(10^2^ − 10^5^) ^c^
**MoSe_2_**	20 (flake) ^d^	3	2.18 ^e^	[n] = 10^18 f^	(20 − 10^4^) ^g^

^a^ Ref. [43]. ^b^ Ref. [44]. ^c^ Refs. [45,46]. ^d^ Ref. [15]. ^e^ Ref. [47]. ^f^ Refs. [48,49]. ^g^ Ref. [50].

## Data Availability

The original contributions presented in this study are included in the article/Supplementary Material. Further inquiries can be directed to the corresponding author.

## Supplementary Materials

The following supporting information can be downloaded at: https://www.mdpi.com/article/10.3390/ma19010204/s1, Figure S1: Comparison between the potential generated by a point charge from the theoretical expression derived by Refs. [35,36] of the manuscript (blue curve (a)) and our approach based on finite element calculations using a 1 nm thin film (green curve, (b)). Inset shows the difference between both curves.; Figure S2: Ez component of the electric field are plotted beyond the MoOx/MoSe_2_ boundary (x = 0), for V_b_ = −15 V and various oxide permittivity. Arrows mark the location of E_z_ maximum. Inset zooms the E_z_ field for the cases of ε_r_ = 100 and 20.

## Abbreviations

The following abbreviations are used in this manuscript:

LAO	Local anodic oxidation
FET	Field effect transistor
MOCVD	Metalorganic chemical vapor deposition
RH	Relative humidity
TMD	Transition metal dichalcogenide

## References

[1] Illarionov Y., Lv Y.Z., Wu Y.H., Chai Y.J. (2024). LAB-to-FAB Transition of 2D FETs: Available Strategies and Future Trends. Nanomaterials.

[2] Yin T., Xu L., Gil B., Merali N., Sokolikova M.S., Gaboriau D.C.A., Liu D.S.K., Mustafa A.N.M., Alodan S., Chen M. (2023). Graphene Sensor Arrays for Rapid and Accurate Detection of Pancreatic Cancer Exosomes in Patients’ Blood Plasma Samples. ACS Nano.

[3] Yu J., Suleiman A.A., Shi J.W., Ong R.J., Ling F.C.-C., Zhang W. (2025). Recent Advances in Atomic Force Microscopy-Based Local Anodic Oxidation Nanolithography of 2D Materials. Adv. Mater. Interfaces.

[4] Cabrera N., Mott N.F. (1949). Theory of the oxidation of metals. Rep. Prog. Phys..

[5] Verwey E.J.W. (1935). Electrolytic conduction of a solid insulator at high fields. The formation of the anodic oxide film on aluminum. Physica.

[6] Dewald J.F. (1955). Theory of the Kinetics of Formation of Anode Films at High Fields. J. Electrochem. Soc..

[7] Fromhold A.T. (1964). Space Charge in Growing Oxide Films. IV. Rate Effects Deduced by an Averaging Technique. J. Chem. Phys..

[8] Dubois E., Bubendorff J.L. (2000). Kinetics of scanned probe oxidation: Space-charge limited growth. J. App. Phys..

[9] Dagata J.A., Inoue T., Itoh J., Matsumoto K., Yokoyama H. (1998). Role of space charge in scanned probe oxidation. J. Appl. Phys..

[10] Avouris P., Hertel T., Martel R. (1997). Atomic force microscope tip-induced local oxidation of silicon: Kinetics, mechanism, and nanofabrication. Appl. Phys. Lett..

[11] Calleja M., Tello M., García R. (2002). Size determination of field-induced water menisci in noncontact atomic force microscopy. J. Appl. Phys..

[12] Ryu Y.K., Garcia R. (2017). Advanced oxidation scanning probe lithography. Nanotechnology.

[13] Garcia R., Martinez R.V., Martinez J. (2006). Nano-chemistry and scanning probe nanolithographies. Chem. Soc. Rev..

[14] Ryu Y.K., Postigo P.A., Garcia F., Garcia R. (2014). Fabrication of sub-12 nm thick silicon nanowires by processing scanning probe lithography masks. Appl. Phys. Lett..

[15] Borodin B.R., Benimetskiy F.A., Alekseev P.A. (2021). Study of local anodic oxidation regimes in MoSe_2_. Nanotechnology.

[16] Lin J.F., Tai C.K., Lin S.L. (2006). Theoretical and experimental studies for nano-oxidation of silicon wafer by ac atomic force microscopy. J. Appl. Phys..

[17] Garcia-Martin A., Garcia R. (2006). Formation of nanoscale liquid menisci in electric fields. Appl. Phys. Lett..

[18] Fan P., Gao J., Mao H., Geng Y., Yan Y., Wang Y., Goel S., Luo X. (2022). Scanning Probe Lithography: State of-the-Art and Future Perspectives. Micromachines.

[19] Snow E.S., Jernigan G.G., Campbell P.M. (2000). The kinetics and mechanism of scanned probe oxidation of Si. Appl. Phys. Lett..

[20] Kinser C.R., Schmitz M.J., Hersam M.C. (2006). Kinetics and Mechanism of Atomic Force Microscope Local Oxidation on Hydrogen-Passivated Silicon in Inert Organic Solvents. Adv. Mater..

[21] Dagata J.A., Perez-Murano F., Abadal G., Morimoto K., Inoue T., Itoh J., Yokoyama H. (2000). Predictive model for scanned probe oxidation kinetics. Appl. Phys. Lett..

[22] Kuramochi H., Pérez-Murano F., Dagata J.A., Yokoyama H. (2004). Faradaic current detection during anodic oxidation of the H-passivated p-Si(001) surface with controlled relative humidity. Nanotechnology.

[23] Kuramochi H., Ando K., Yokoyama H. (2003). Effect of humidity on nano-oxidation of p-Si(0 0 1) surface. Surf. Sci..

[24] Filipovic L., Selberherr S. (2013). A method for simulating Atomic Force Microscope nanolithography in the Level Set framework. Microelectron. Eng..

[25] Dagata J.A., Perez-Murano F., Martin C., Kuramochi H., Yokoyama H. (2004). Current, charge, and capacitance during scanning probe oxidation of silicon. I. Maximum charge density and lateral difusión. J. Appl. Phys..

[26] Calleja M., García R. (2000). Nano-oxidation of silicon surfaces by noncontact atomic-force microscopy: Size dependence on voltage and pulse duration. Appl. Phys. Lett..

[27] Luo X., Gao J., Xie W., Hasan R.M.M., Qin Y. (2023). Flexible single-step fabrication of programmable 3D nanostructures by pulse-modulated local anodic oxidation. CIRP Ann..

[28] Quesada S.J., Borrás F., García-Vélez M., Coya C., Climent E., Munuera C., Villar I., de la Peña O’Shea V.A., de Andrés A., Alvarez A.L. (2019). New Concepts for Production of Scalable Single Layer Oxidized Regions by Local Anodic Oxidation of Graphene. Small.

[29] Alvarez A.L., Coya C., García-Vélez M. (2015). Development of electrical-erosion instrument for direct write micropatterning on large area conductive thin films. Rev. Sci. Instrum..

[30] Lee C., Wei X., Kysar J.W., Hone J. (2008). Measurement of the Elastic Properties and Intrinsic Strength of Monolayer Graphene. Science.

[31] *COMSOL Multiphysics^®^*, v. 4.4. https://www.comsol.com.

[32] García R., Calleja M., Pérez-Murano F. (1998). Local oxidation of silicon surfaces by dynamic force microscopy: Nanofabrication and water bridge formation. Appl. Phys. Lett..

[33] Gomer R. (1986). Extensions of the Field-Emission Fluctuation Method for the Determination of Surface Diffusion Coefficients. Appl. Phys. A..

[34] Bartošík M., Škoda D., Tomanec O., Kalousek R., Jánský P., Zlámal J., Spousta J., Dub P., Šikola T. (2009). Role of humidity in local anodic oxidation: A study of water condensation and electric field distribution. Phys. Rev. B.

[35] Cudazzo P., Tokatly I.V., Rubio A. (2011). Dielectric screening in two-dimensional insulators: Implications for excitonic and impurity states in graphene. Phys. Rev. B.

[36] Keldysh L.V. (1979). Coulomb interaction in thin semiconductor and semimetal films. J. Exp. Theor. Phys. Lett..

[37] Yao Y., Ren L., Gao S., Li S. (2017). Histogram method for reliable thickness measurements of Graphene films using atomic force microscopy (AFM). J. Mater. Sci. Technol..

[38] Shearer C.J., Slattery A.D., Stapleton A.J., Shapter J.G., Gibson C.T. (2016). Accurate thickness measurement of Graphene. Nanotechnology.

[39] Bu T., Gao H., Yao Y., Wang J., Pollard A.J., Legge E.J., Clifford C.A., Delvallée A., Ducourtieux S., Lawn M.A. (2023). Thickness measurements of graphene oxide flakes using atomic force microscopy: Results of an international interlaboratory comparison. Nanotechnology.

[40] Jaejun Park J., Lee W., Nam J., Han J.T., Choi C.-J., Hwang J.Y. (2022). A study of the correlation between the oxidation degree and thickness of graphene oxides. Carbon.

[41] Cowie M., Plougmann R., Benkirane Y., Schué L., Schumacher Z., Grütter P. (2022). How high is a MoSe_2_ monolayer?. Nanotechnology.

[42] Ryu Y.K., Dago A.I., He Y., Espinosa F.M., Lopez-Elvira E., Munuera C., Garcia R. (2021). Sub-10 nm patterning of few-layer MoS2 and MoSe2 nanolectronic devices by oxidation scanning probe lithography. Appl. Surf. Sci..

[43] Gao W., Xiao P., Henkelman G., Liechti K.M., Huang R. (2014). Interfacial adhesion between graphene and silicon dioxide by density functional theory with van der Waals corrections. J. Phys. D Appl. Phys..

[44] Properties of the Commercial Graphene Layers Transferred onto Quartz Used Here. https://www.graphenea.com/collections/buy-graphene-films/products/monolayer-graphene-on-quartz-4-wafer?variant=51790150035.

[45] Liu J., Galpaya D., Notarianni M., Yan C., Motta N. (2013). Graphene-based thin film supercapacitor with graphene oxide as dielectric spacer. Appl. Phys. Lett..

[46] Kumar K.S., Pittala S., Sanyadanam S., Paik P. (2015). A new single/few-layered graphene oxide with a high dielectric constant of 1e6: Contribution of defects and functional groups. RSC Adv..

[47] Ugeda M.M., Bradley A.J., Shi S.-F., da Jornada F.H., Zhang Y., Qiu D.Y., Ruan W., Mo S.-K., Hussain Z., Shen Z.-X. (2014). Giant bandgap renormalization and excitonic effects in a monolayer transition metal dichalcogenide semiconductor. Nat. Mater..

[48] Qiu C., Zhang C., Geng S., Wang F., Deng H.X. (2022). First-Principles Study of the Origin of the Distinct Conductivity Type of Monolayer MoSe_2_ and WSe_2_. J. Phys. Chem. C.

[49] Jin Y., Keum D.H., An S.J., Kim J., Lee H.S., Lee Y.H. (2015). A Van Der Waals Homojunction: Ideal p–n Diode Behavior in MoSe_2_. Adv. Mater..

[50] Afanasiev P., Lorentz C. (2019). Oxidation of Nanodispersed MoS_2_ in Ambient Air: The Products and the Mechanistic Steps. J. Phys. Chem. C.

[51] Díez-Betriu X., Álvarez-García S., Botas C., Álvarez P., Sánchez-Marcos J., Prieto C., Menéndez R., de Andrés A. (2013). Raman spectroscopy for the study of reduction mechanisms and optimization of conductivity in graphene oxide thin films. J. Mater. Chem. C.

[52] Alkhouzaama A., Qiblaweya H., Khraisheha M., Atiehb M., Al-Ghouti M. (2020). Synthesis of graphene oxides particle of high oxidation degree using a modified Hummers method. Ceram. Int..

[53] Hong S., Im H., Hong Y.K., Liu N., Kim S., Park J.H. (2018). n-Type Doping Effect of CVD-Grown Multilayer MoSe_2_ Thin Film Transistors by Two-Step Functionalization. Adv. Electron. Mater..

[54] Kang S., Khan H., Lee C., Kwon K., Lee C.S. (2021). Investigation of hydrophobic MoSe_2_ grown at edge sites on TiO_2_ nanofibers for photocatalytic CO_2_ reduction. Chem. Eng. J..

[55] Uhlig M.R., Martin-Jimenez D., Garcia R. (2019). Atomic-scale mapping of hydrophobic layers on graphene and few-layer MoS_2_ and WSe_2_ in water. Nat. Commun..

[56] Ramos-Alvarado B., Kumar S., Peterson G.P. (2016). On the wettability transparency of graphene-coated silicon surfaces. J. Chem. Phys..

[57] Choi S.Y., Kim S., Kim Y., Park M., Son M.G., Ma J.Y., Park Y.M., Kim J.H., Kang H., Kim J. (2025). Wetting Transparency-Induced Enhancement of Moisture Stability in Monolayer Transition Metal Dichalcogenides. Small.

[58] Ryu Y.K., Knoll A.W., Celano U. (2019). Oxidation and Thermal Scanning Probe Lithography for High-Resolution Nanopatterning and Nanodevices. Electrical Atomic Force Microscopy for Nanoelectronics.

[59] Sygellou L., Paterakis G., Galiotis C., Tasis D. (2016). Work Function Tuning of Reduced Graphene Oxide Thin Films. J. Phys. Chem. C.

