# Supplementary material for: Modeling the Dynamics of Electric Field-Assisted Local Functionalization in Two-Dimensional Materials
Source: Materials (Basel). 2026 Jan 5;19(1):204. doi: 10.3390/ma19010204 (PMC12787140; doi:10.3390/ma19010204)
Supplement: Supplementary file 1 [file materials-19-00204-s001.zip › materials-4012376-supplementary.pdf]

materials

MDPI

Electronic Supplementary Information

Modelling the Dynamics of Electric Field-Assisted Local Functionalization in Two-Dimensional Materials

Fernando Borrás [1], Julio Ramiro-Bargueño [1], Óscar Casanova-Carvajal [2,3] Alicia de Andrés [4], Sergio J. Quesada [5] and Ángel Luis Álvarez [1,*]

[1] Escuela de Ingeniería de Fuenlabrada, Universidad Rey Juan Carlos, Fuenlabrada (Madrid), 28942, Spain; f.borras.2019@alumnos.urjc.es , julio.ramiro@urjc.es , angelluis.alvarez@urjc.es
[2] Centro de Tecnología Biomédica, Campus de Montegancedo, Universidad Politécnica de Madrid, Madrid, Spain; oscar.casanova@ctb.upm.es
[3] Departamento de Ingeniería Eléctrica, Electrónica, Automática y Física Aplicada, Escuela Técnica Superior de Ingeniería y Diseño Industrial ETSIDI, Universidad Politécnica de Madrid, 28040 Madrid, Spain;;
[4] Instituto de Ciencia de Materiales de Madrid, Consejo Superior de Investigaciones Científicas, Cantoblanco, Madrid, 28049, Spain; ada@icmm.csic.es
[5] Independent researcher; quesada@quesada-consulting.es
* Correspondence: angelluis.alvarez@urjc.es ;

List of contents

1. Comparison between the perfect mathematical solution for the electrostatic potential in a 2D material, and a thin film approach 2

2. Some details on the structures represented in Figure 1 of the manuscript.. 2

3. Data for the expansion of the oxidized region over time in MoSe2 platelets. 6

1. Comparison between the Perfect Mathematical Solution for the Electrostatic Potential in a 2D Material, and a Thin Film Approach.

It is important to note that it has been theoretically demonstrated the equivalence between the potential resulting from an elementary charge in a pure 2D dielectric sheet of polarizability α, with air above and below, and that derived for a thin film Refs. 35 and 36 of the manuscript. In both cases, the generated potential no longer obeys Coulomb law but a combination of Struve and 2nd order Bessel functions. This is because in either a pure 2D insulator or a thin film the macroscopic charge screening due to the material polarizability cannot be described by a single dielectric constant that renormalizes the electronic charge, as in 3D systems (Ref. 35 of the manuscript)

To validate the approach of using a large thin film of 1 nm thickness as a 2D layer, we have created a model using a disk (1 nm thick, with a radius orders of magnitude longer, up to 30 microns) at the axis of which we have placed a small vertical segment 1 nm long of elementary charge. The potential derived from the analytical solution proposed in Refs 35, 36 of the manuscript, together with our electrostatic results obtained by finite element calculations are plotted (Figure S1) for comparison up to a long distance

from the central charge, including the normalized error, and revealing the excellent agreement between theory and simulation.

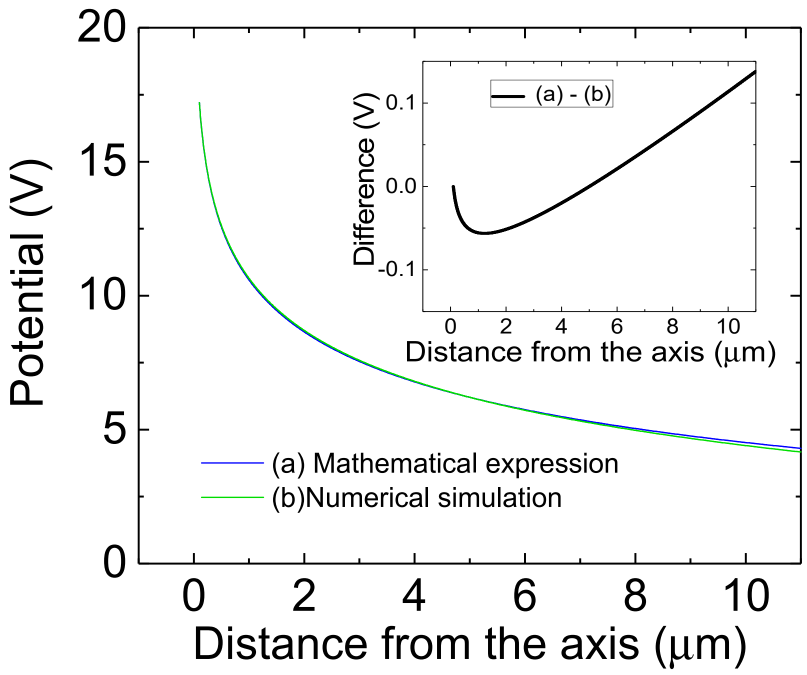

Figure S1. Comparison between the potential generated by a point charge from the theoretical expression derived by Refs. 35, 36 of the manuscript (blue curve (a)) and our approach based on finite element calculations using a 1 nm thin film (green curve, (b)). Inset shows the difference between both curves.

2. Some Details on the Structures Represented in Figure 1 of the Manuscript.

According to a typical experimental setup consisting of probe and sample, the selected test structures have radial symmetry around the probe (*z*-axis). Two geometries representing different real cases in Refs.15, 28 of the manuscript, have been tested: both include a 2–3 nm thick layer of insulating oxide in the central circular region (around the *z*-axis), surrounded by a 1 nm thick graphene layer (Figure 1a) or 20 nm thick layer in case of $MoSe_2$ (Figure 1b). Although the latter is not a 2D layer, it represents those samples where oxide expansion data are available. It serves as an example of the validity of our approach for thin layers in general. On the upper part of the oxide layers, an insulating wetting layer with dielectric properties of pure water (relative permittivity $\varepsilon_r = 80$) is placed. We note that such a continuous layer is consistent with high values of relative humidity, typically RH > 80%. For low values RH < 40%, the promotion of a water meniscus below the probe indeed determines the region where oxidation is more favored. For intermediate values of RH, the validity of the approach of reducing the average dielectric permittivity is under study.

On top of this structure, a central conductor disc of radius like those in experiments (6 μm in graphene, and 10 nm for TMDCG samples) represents the metal probe contact as

that reported experimentally in Refs.15, 28 of the manuscript. For simplicity the probe is considered in contact with the sample.

3. Vertical Component of the Electric Field (Ez) Close to the Boundary Mo⚜x/MoSe2, in MoSe2 Platelets, for Different Permittivity.

Figure S2 shows the E_z component of the field generated at a bias voltage $V_b = -15V$ along a radius up to 25 nm from the boundary $MoO_x/MoSe_2$, for various oxide permittivity ranging from 20 to 10^5. As permittivity decreases below $\varepsilon_r \approx 10^3$ there is a drastic reduction in field intensity, about two orders of magnitude. Additionally, there is a pronounced broadening, a shift away of the E_z maximum, and appears a negative E_z value (resulting from insufficient screening) precisely at the boundary, which is inconsistent with attachment of OH^- species to it. So a reduction of the permittivity as described in Ref. 51 of the manuscript would indeed cause a quick deceleration of the oxide expansion. Thus, although not included in our current simulations, a slowdown in radius expansion is expected due to a rapid evolution of oxide species from those with high polarizability (compatible with permittivity close to 10^3) to MoO_x compounds, with a low permittivity, similarly as observed for ambient conditions in Ref. 51.

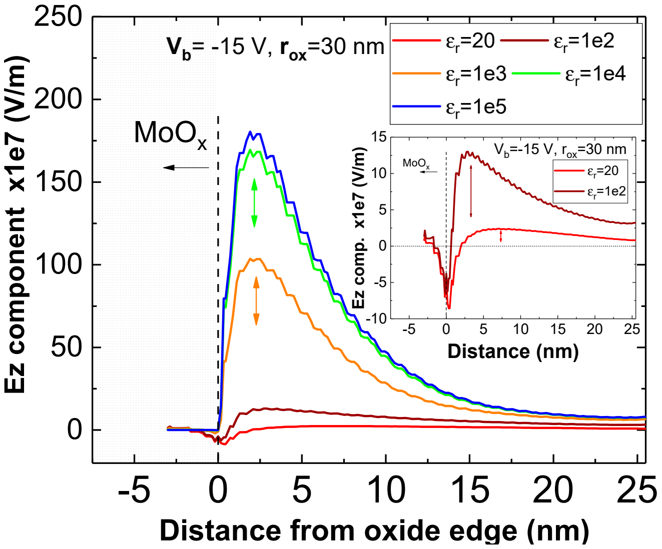

Figure S2. Ez component of the electric field is plotted beyond the MoOx/$MoSe_2$ boundary (x = 0), for $V_b = -15V$ and various oxide permittivity. Arrows mark the location of E_z maximum. Inset zooms the E_z field for the cases of $\varepsilon_r = 100$ and 20.
